# Additions to The Knowledge of *Tubakia* (*Tubakiaceae*, *Diaporthales*) in China

**DOI:** 10.3390/jof8111143

**Published:** 2022-10-28

**Authors:** Ya-Quan Zhu, Ning Jiang, Zhi-Peng Dou, Han Xue, Chun-Gen Piao, Yong Li

**Affiliations:** 1 Key Laboratory of Forest Protection of National Forestry and Grassland Administration, Ecology and Nature Conservation Institute, Chinese Academy of Forestry, Beijing 100091, China; 2 Beijing Key Laboratory for Forest Pest Control, Beijing Forestry University, Beijing 100083, China; 3 Chinese Academy of Forestry, Beijing 100091, China

**Keywords:** *Diaporthales*, multigene phylogeny, *Quercus*, *Tubakiaceae*

## Abstract

The species of *Tubakia* (*Tubakiaceae, Diaporthales*, *Sordariomycetes*) have often been reported as endophytes and pathogens on woody plants. During the investigation of *Tubakia* species from *Fagaceae* trees in China, 46 isolates were obtained from diseased leaves and seeds. The characterization of these isolates was based on the observation of morphological characters, the effect of temperature on mycelial growth rate, as well as the combined genes of ITS, *tef1* and *tub2*. As a result, six species were identified: *Tubakia americana*, *T. cyclobalanopsidis* sp. nov., *T. dryinoides*, *T. koreana*, *T. paradryinoides* and *T. quercicola* sp. nov. Among these, *T. koreana and T. paradryinoides* were firstly discovered in China. Pathogenicity tests were conducted using the conidial suspension on young, excised leaves for these six species, which showed that they were mildly pathogenic to four Fagacece hosts: *C. mollissima*, *Q. acutissima*, *Q. aliena* var. *acutiserrata* and *Q. variabilis*.

## 1. Introduction

The genus *Tubakia* (*Tubakiaceae*, *Diaporthales*, *Sordariomycetes*) is introduced based on the type species *T. japonica* [1,2,3]. *Tubakia* is characterized by pycnothyrial conidiomata composed of convex scutella with cells fixed to the substratum by a central columella. The conidia are globose, subglobose, ellipsoid, teardrop to subcylindrical or irregular shape, aseptate, hyaline, subhyaline to pigmented [1,4,5]. Additionally, several species (*T*. *americana*, *T*. *dryina*, *T*. *dryinoides*, *T*. *hallii*, *T*. *iowensis*, *T*. *japonica*, *T*. *macnabbii*, *T*. *melnikiana*, *T*. *sierrafriensis*, *T*. *suttoniana*) can produce a second type of much smaller conidia, named microconidia [1].

Saccardo introduced the genus *Actinopelte* with *A. japonica* as the type [2]. Subsequently, Höhnel added *A*. *americana* and *A*. *dryina* to this genus [6]. Yokoyama and Tubaki described *A*. *castanopsidis*, *A*. *rubra* and *A*. *subglobosa* according to comprehensive examinations based on Japanese collections [7]. Since *Actinopelte* turned out to be illegitimate (later homonym of *Actinopelte* Stitzenb. 1861), Sutton introduced the alternative name *Tubakia* [1,2,3]. Several species were revealed from leaves of *Quercus* spp. in the USA, namely *T. hallii*, *T. macnabbii* and *T. tiffanyae* [3]. Braun revised this genus based on morphological and phylogenetic data, and *Tubakia* was expanded as a family *Tubakiaceae* [1]. Subsequently, five additional species named *T*. *californica*, *T*. *melnikiana*, *T*. *oblongispora*, *T. paradryinoides* and *T*. *sierrafriensis* were introduced based on in vivo and in vitro morphological analyses, as well as phylogenetic data [1]. In addition, *T. koreana* and *T. lushanensis* were described from China and Korea, respectively [8,9]. Until now, a total of 19 species have been accepted into this genus.

Species of *Tubakia* have been mainly reported as endophytes in leaves and twigs, and pathogens in leaf spots, blotch and necrosis [1,8,10,11,12,13,14]. Nearly all *Tubakia* species were reported from fagaceous hosts, including species of plant genera *Castanea*, *Castanopsis*, *Fagus*, *Lithocarpus* and *Quercus*. Additionally, there are some reports on the other host families, i.e., *Altingiaceae*, *Anacardiaceae*, *Nyssaceae*, *Oleaceae*, *Sapindaceae* and *Ulmaceae* [1]. Members of *Tubakia* have been reported in America, China, Japan, Korea and Europe [5,7,8,9,10,11,12,13,14,15,16,17,18,19,20,21].

In Japan, Matsumura et al. examined the endophyte communities of seven evergreen *Quercus* species, showing that host identity and ecology were significantly associated with *Tubakia* community structure [20]. *T*. *iowensis* as a serious pathogen causes necrosis of the leaf tissue along the veins and the eventual death of entire leaves on *Q. macrocarpa* in North America [11,12]. *T*. *dryina* was discovered as leaf pathogens of *Fagus sylvatica*, *Quercus robur* and *Tilia cordata* in Poland [10].

In China, six species of *Tubakia* have been reported, *viz*. *T. americana* on seeds of *Quercus variabilis* [16], *T*. *chinensis* on *Castanea henryi* [15], *T. dryina* from *Quercus* spp. and *Castanea* spp. [13], *T. japonica* on *Castanea mollissima* [18], *T*. *lushanensis* from leaves of *Quercus palustris* [9] and *T*. *seoraksanensis* on *Quercus mongolica* [17]. The present study describes two novel species and four known species of *Tubakia* in China based on both morphology and phylogeny.

## 2. Materials and Methods

### 2.1. Isolation and Morphological Characterization

From 2018 to 2020, specimens were collected during investigations for plant diseases in Mount Huang (Huangshan City), Shushan Forest Park (Hefei City) and Zipeng Mountain (Hefei City) of Anhui Province; Guangzhou City of Guangdong Province; Kuankuoshui national nature reserve (Zunyi City) of Guizhou Province; Kikunshan National Nature Reserve (Xinyang City) and Yaoshan Mountain (Pingdingshan City) of Henan Province; Foping County (Hanzhong City), Panjiawan Forest Park (Baoji City) and Zhuque National Forest Park (Xian City) of Shaanxi province. Isolates of *Tubakia* in this study were obtained from diseased leaves and seeds of *C. mollissima*, *Q. acutissima*, *Q. aliena* var. *acuteserrata*, *Q. glauca* and *Q. variabilis*. 

The leaf and seed samples were first surface-sterilised for 1 min in 75% ethanol, 3 min in 1.25% sodium hypochlorite and 1 min in 75% ethanol, rinsed for 2 min in distilled water and blotted on dry sterile filter paper. Then, the diseased areas of the leaves were cut into 0.5 × 0.5 cm pieces using an aseptic razor blade, transferred on to the surface of potato dextrose agar plates (PDA; 200 g potatoes, 20 g dextrose, 20 g agar per litre) and incubated at 25 °C to obtain pure cultures. The cultures were deposited in the China Forestry Culture Collection Center (CFCC; http://cfcc.caf.ac.cn/) and the specimen was deposited in the Herbarium of the Chinese Academy of Forestry (CAF; http://museum.caf.ac.cn/).

To determine the effect of temperature on mycelial growth and the optimal growth temperature, the representative isolates were cultured on PDA and malt extract agar (MEA, 30 g malt extract, 5 g mycological peptone, 15 g agar per litre) for further assays. After seven days of incubation at 25 °C, 5 mm diam. mycelial plugs were transferred from the edge of the colonies to the center Petri plates. The plates were incubated at 5–10–15–20–25–30–35–40 °C in the dark. Three Petri plates were used for each temperature as replicates. 

Microscopic structures of the fungus growing on a medium were mounted in water and examined under an Axio Imager 2 microscope (Zeiss, Oberkochen, Germany). At least 30 measurements were made for each structure examined.

### 2.2. DNA Extraction, Amplification and Sequencing 

Genomic DNA was extracted from the fresh mycelium harvested from PDA plates after 4 days using a cetyltrimethylammonium bromide (CTAB) method [22]. For initial species confirmation, the internal transcribed spacer (ITS) region was sequenced for all isolates. The BLAST tool (https://blast.ncbi.nlm.nih.gov/Blast.cgi, 15 August 2022) was used to compare the resulting sequences with those in GenBank (Table A1). After confirmation of *Tubakia* species, two additional gene regions coding for translation elongation factor 1-alpha (*tef1*), and beta-tubulin (*tub2*) were sequenced. Three loci were amplified with the following primer pairs, ITS1 and ITS4 for ITS [23], T1 and 688F for *tef1* [24], and T1/Bt2a and Bt2b for *tub2* [25,26]. The primer pairs and amplification conditions for each of the above-mentioned gene regions are provided in Table 1.

A PCR reaction was conducted in a 20 µL reaction volume, and the components were as follows: 1 µL DNA template (20 ng/μL), 1 µL forward 10 µM primer, 1 µL reverse 10 µM primer, 10 µL T5 Super PCR Mix (containing Taq polymerase, dNTP and Mg^2+^, Beijing Tisingke Biotech Co., Ltd., Beijing, China), and 7 µL sterile water. Amplifications were performed using a T100 Thermal Cycler (Bio-Rad, Hercules, CA, USA). Strands were sequenced in both directions using PCR primers. All amplified PCR products were estimated visually with 1.4% agarose gels stained with ethidium bromide and then PCR positive products were sent to Sangon Biotech (Shanghai) Co., Ltd., (Beijing, China) for sequencing. The new sequences generated in this study, as well as the reference sequences of all isolates used in the present study, are listed in Table 2.

### 2.3. Phylogeny

The sequences generated in this study were supplemented with additional sequences obtained from GenBank (Table 2). The dataset consisted of 94 sequences, including one outgroup taxon, *Melanconis groenlandica* (CBS 116540). The sequences were aligned with the MAFFT v.7, after which the alignments were manually corrected using MEGA v.7.0. [27,28]. Phylogenetic analyses, including Maximum Likelihood (ML) and Bayesian Inference (BI) methods, were conducted for the single gene sequence data sets of the ITS, *tef1* and *tub2*, and the combined data set of all three gene regions. ML analyses were conducted using RAxML-HPC BlackBox 8.2.10 on the CIPRES Science Gateway portal (https://www.phylo.org, 12 June 2022), employing a GTRGAMMA substitution model with 1000 bootstrap replicates [29,30]. Bayes inference was conducted using a Markov Chain Monte Carlo (MCMC) algorithm in MrBayes v.3.0 [31]. Two MCMC chains were run from a random starting tree for 1,000,000 generations, resulting in a total of 10,000 trees. The first 25% of trees sampled were discarded as burn-in, and the remaining trees were used to calculate the posterior probabilities. Branches with significant Bayesian Posterior Probabilities (BPP > 0.9) were estimated in the remaining 7500 trees. Phylogenetic trees were viewed with FigTree v.1.3.1 and processed by Adobe Illustrator CS5.

### 2.4. Pathogenicity Test

The isolates of each *Tubakia* species were used for a pathogenicity test on four hosts, *viz*. *C*. *mollissima*, *Q. acutissima*, *Q. aliena* var. *acuteserrata* and *Q*. *variabilis*. Each isolate was incubated on PDA for 7–15 days at 25 °C to achieve spore suspension. Fresh leaves without visible diseases were collected from 1-year-old *Fagaceae* plants and used for the tests. These leaves were surface sterilized in 75% ethanol for 3 min and 1% sodium hypochlorite for 3 min, and then rinsed thrice in sterile distilled water.

After washing and air drying, these leaves (six of each species) were surface wounded by a sterile needle, and 10 µL of conidial suspension (1 × 10^6^ conidia/mL) was inoculated on the wounds. Sterile water was used as a control treatment. Inoculated leaves were placed in glass containers on top of moist paper and sealed. The containers were placed in a growth chamber and incubated at 25 °C with an alternation of 12 h of light and 12 h of darkness for 14 days. Symptom development of the leaves was checked daily and recorded for up to 14 days.

### 2.5. Statistical Analyses

Regression analyses were applied to the means of all independent quantitative variables. Nonlinear regression models were evaluated for describing the relationship between mycelial growth and temperature. The Gram-Charlier A series (GCAS) was selected because it provided a good fit for all isolates. All these analyses were conducted using OriginPro 2018 [32]. GCAS were fitted to the values of mycelial growth versus temperature for each isolate, and the optimum temperatures were calculated in the fitted equations. 

## 3. Results

### 3.1. Phylogenetic Analyses

**Figure 1:**
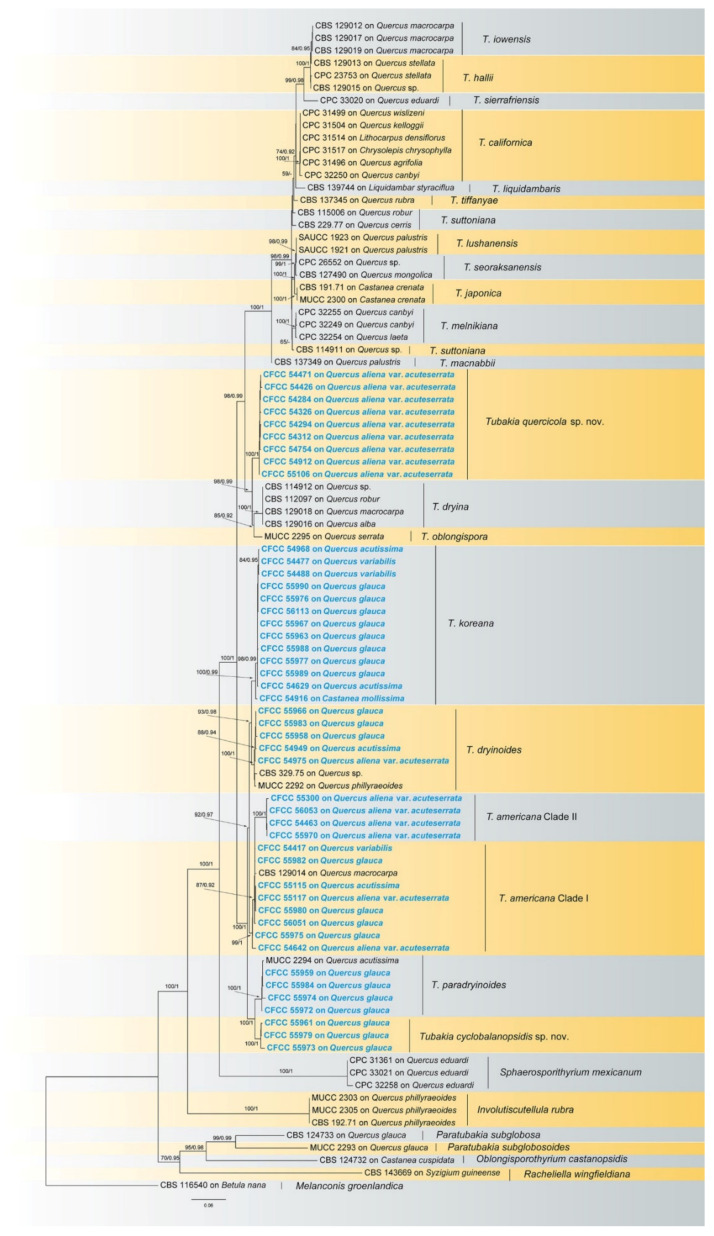
A phylogram of *Tubakia* resulting from a maximum likelihood analysis based on a combined matrix of ITS, *tef1* and *tub2*. Numbers above the branches indicate ML bootstrap values (left, ML BS ≥ 50%) and Bayesian Posterior Probabilities (right, BPP ≥ 0.9). The tree is rooted with *Melanconis groenlandica* (CBS 116540). Isolates from the present study are marked in blue and bold face.

The combined three-gene sequence dataset (ITS, *tef1* and *tub2*) was analysed to determine the phylogenetic position of the new isolates obtained in this study. A total of 1919 characters, including gaps (686 for ITS, 665 for *tef1* and 568 for *tub2*), were included in the dataset used in the phylogenetic analyses. Of these characters, 1024 were constant, 218 were variable, but parsimony-uninformative and 677 were parsimony-informative. The best ML tree (lnL =−13,482.15) revealed by RAxML is shown as a phylogram in Figure 1. The topologies resulting from ML and BI analyses were congruent (Figure 1). Isolates in the present study were separated into seven supported clades in *Tubakia*, with two new clades representing two new species. Nine isolates named CFCC 54471, CFCC 54426, CFCC 54284, CFCC 54326, CFCC 54294, CFCC 54312, CFCC 54754, CFCC 54912 and CFCC 55106 formed a well-supported clade representing a novel species *Tubakia quercicola* close to *T*. *dryina. Tubakia cyclobalanopsidis* sp. nov. (CFCC 55961, CFCC 55979 and CFCC 55973) grouped sister with *T. paradryinoides.*

### 3.2. Morphology

**Figure 2:**
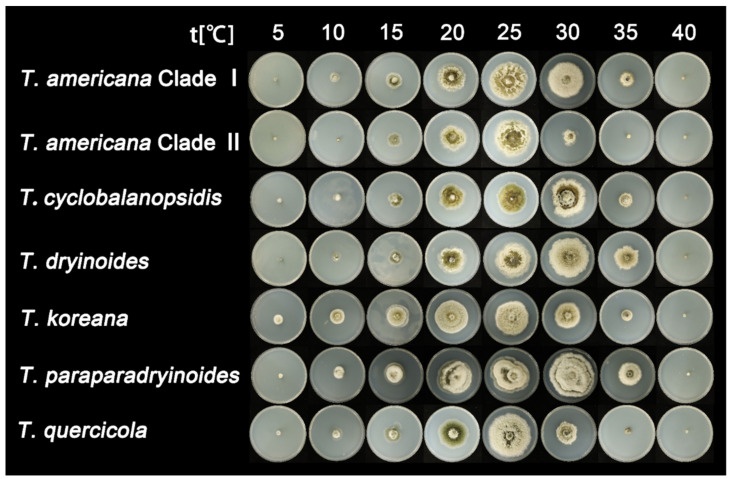
The effect of temperature on the mycelial growth rate of six *Tubakia* species on PDA after seven days of incubation.

**Figure 3:**
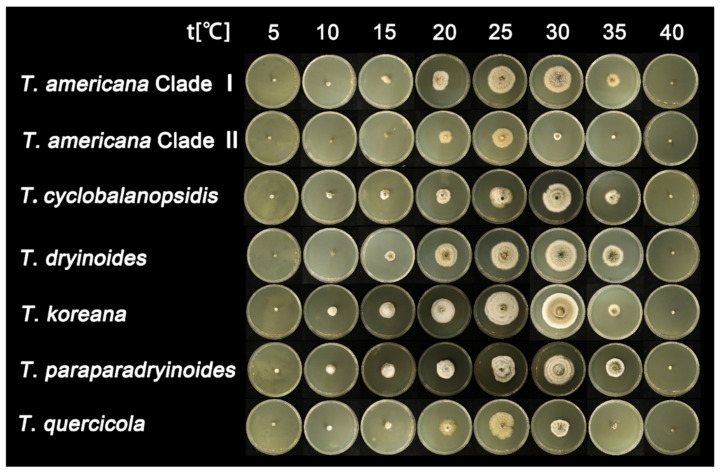
The effect of temperature on the mycelial growth rate of six *Tubakia* species on MEA after seven days of incubation.

After seven days of incubation, no mycelial growth was observed at 40 °C. *Tubakia quercicola* grows faster than the other species (0.73 cm/day) on PDA at 25 °C, whereas *Tubakia cyclobalanopsidis* grows the slowest (0.54 cm/day). Furthermore, the rate of colonies growth on PDA is faster than those on MEA. The growth ranges and growth rate of each temperature are significant between *T. americana* clade I and *T. americana* clade II. All results of the effects of temperature on mycelial growth rate are shown in Table 3 and Figure 2 and Figure 3.

### 3.3. Statistical Analyses

**Figure 4:**
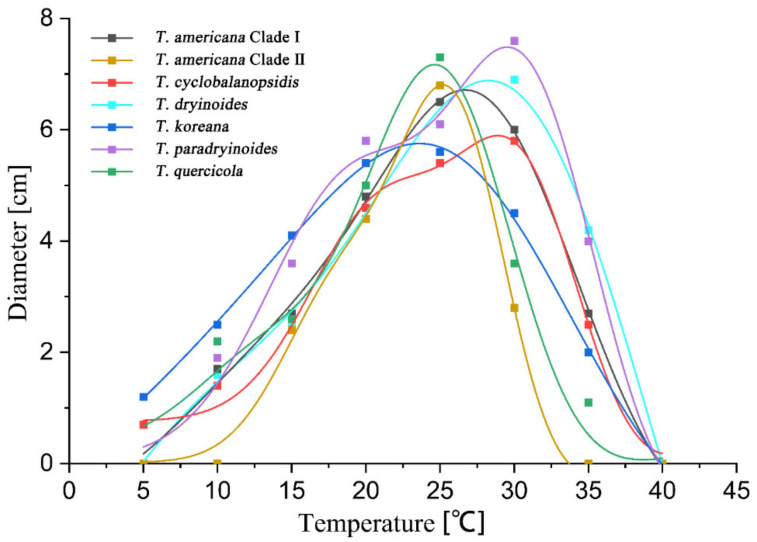
The effect of temperature on the mycelial growth rate of six *Tubakia* species on PDA. The averages of radial growth rate and temperatures were adjusted to a nonlinear regression curve through the Analytis GCAS model. Data points are the means of two independent experiments of three replicated Petri dishes. The formula of the nonlinear fitting curve is as follows: *T. americana* clade I = −1.00 + 6.89 × exp(−((x − 25.53)/8.13)^2^/2) × (((x − 25.53)/8.13)^3^ − 3 × ((x − 25.53)/8.13)) + 0.02 × (((x − 25.53)/8.13)^4^ − 6 × ((x − 25.53)/8.13)^3^ + 3)), R^2^ = 0.99669; *T. americana* clade II = 0.02 + 5.89 × exp(−((x − 24.68)/4.44)^2^/2) × (((x − 24.68)/4.44)^3^ − 3 × ((x − 24.68)/4.44)) + 0.03 × (((x − 24.68)/4.44)^4^ − 6 × ((x − 24.68)/4.44)^3^ + 3)), R^2^ = 0.99555; *T. cyclobalanopsidis* = 0.76 + 4.27 × exp(−((x − 29.09)/5.45)^2^/2) × (((x − 29.09)/5.45)^3^ − 3 × ((x − 29.09)/5.45)) + 0.07 × (((x − 29.09)/5.45)^4^ − 6 × ((x − 29.09)/5.45)^3^ + 3)), R^2^ = 0.99431; *T. dryinoides* = −6.25 + 11.19 × exp(−((x − 26.78)/11.98)^2^/2) × (((x − 26.78)/11.98)^3^ − 3 × ((x − 26.78)/11.98)) + 0.07 × (((x − 26.78)/11.98)^4^ − 6 × ((x − 26.78)/11.98)^3^ + 3)), R^2^ = 0.99412; *T. koreana* = −1.49 + 6.93 × exp(−((x − 24.06)/10.41)^2^/2) × (((x − 24.06)/10.41)^3^ − 3 × ((x − 24.06)/10.41)) + 0.02 × (((x − 24.06)/10.41)^4^ − 6 × ((x − 24.06)/10.41)^3^ + 3)), R^2^ = 0.99901; *T*. *paradryinoides* = 0.07 + 5.94 × exp(−((x − 28.84)/6.49)^2^/2) × (((x − 28.84)/6.49)^3^ − 3 × ((x − 28.84)/6.49)) + 0.06 × (((x − 28.84)/6.49)^4^ − 6 × ((x − 28.84)/6.49)^3^ + 3)), R^2^ = 0.98994; *T*. *quercicola* = 0.27 + 5.4 × exp(−((x − 22.76)/5.92)^2^/2) × (((x − 22.76)/5.92)^3^ − 3 × ((x − 22.76)/5.92)) + 0.01 × (((x − 22.76)/5.92)^4^ − 6 × ((x − 22.76)/5.92)^3^ + 3)), R^2^ = 0.97306.

**Figure 5:**
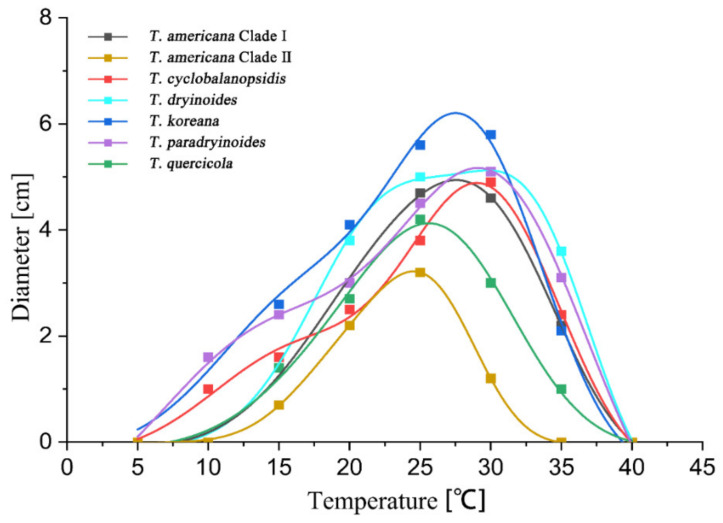
The effect of temperature on the mycelial growth rate of six *Tubakia* species on MEA. The averages of radial growth rate and temperatures were adjusted to a nonlinear regression curve through the Analytis GCAS model. Data points are the means of two independent experiments of three replicated Petri dishes. The formula of the nonlinear fitting curve is as follows: *T*. *americana* clade I = −0.15 + 4.6 × exp(−((x − 30.62)/6.44)^2^/2) × (((x − 30.62)/6.44)^3^ − 3 × ((x − 30.62)/6.44)) + 0.58 × (((x − 30.62)/6.44)^4^ − 6 × ((x − 30.62)/6.44)^3^ + 3)), R^2^ = 0.99713; *T*. *americana* clade II = −0.01 + 3.1 × exp(−((x − 25.27)/4.28)^2^/2) × (((x − 25.27)/4.28)^3^ − 3 × ((x − 25.27)/4.28)) + 0.03 × (((x − 25.27)/4.28)^4^ − 6 × ((x − 25.27)/4.28)^3^ + 3)), R^2^ = 0.99971; *T*. *cyclobalanopsidis* = −0.28 + 3.92 × exp( − ((x − 26.98)/6.98)^2^/2) × (((x − 26.98)/6.98)^3^ − 3 × ((x − 26.98)/ 6.98)) + 0.02 × (((x − 26.98)/6.98)^4^ − 6 × ((x − 26.98)/6.98)^3^ + 3)), R^2^ = 0.99557; *T*. *dryinoides* = −0.13 + 4.38 × exp(−((x − 31.77)/6.17)^2^/2) × (((x − 31.77)/6.17)^3^ − 3 × ((x − 31.77)/6.17)) + 0.09 × (((x − 31.77)/6.17)^4^ − 6 × ((x − 31.77)/6.17)^3^ + 3)), R^2^ = 0.99826; *T*. *koreana* = −0.21 + 5.41 × exp(−((x − 26.39)/6.81)^2^/2) × (((x − 26.39)/6.81)^3^ − 3 × ((x − 26.39)/6.81)) + 0.03 × (((x − 26.39)/6.81)^4^ − 6 × ((x − 26.39)/6.81)^3^ + 3)), R^2^ = 0.99284; *T*. *paradryinoides* = −1.63 + 5.48 × exp( − ((x − 27.50)/8.84)^2^/2) × (((x − 27.50)/8.84)^3^ − 3 × ((x − 27.50)/8.84)) + 0.40 × (((x − 27.50)/8.84)^4^ − 6 × ((x − 27.50)/8.84)^3^ + 3)), R^2^ = 0.9985; *T*. *quercicola* = −0.14 + 4.21 × exp(−((x − 26.57)/6.01)^2^/2) × (((x − 26.57)/6.01)^3^ − 3 × ((x − 26.57)/6.01)) + 0.02 × (((x − 26.57)/6.01)^4^ − 6 × ((x − 26.57)/6.01)^3^ + 3)), R^2^ = 0.99224.

The highest growth rate on PDA of *T. americana* clade I, *T. americana* clade II, *T. koreana* and *T*. *quercicola* were observed at 25 °C. The Analytis GCAS model shows that the optimal growth temperature of these four species is 26.7 °C, 25.2 °C, 23.6 °C and 24.6 °C, respectively. The highest growth rate on PDA for *T. cyclobalanopsidis*, *T. dryinoides* and *T. paradryinoides* were observed at 30 °C, and the Analytis GCAS model shows that the optimal growth temperature is 28.3 °C, 28.9 °C and 29.5 °C, respectively. All results of the effects of temperature on the mycelial growth rate are shown in Figure 4 and Figure 5 and in Table 3. 

### 3.4. Taxonomy

**Figure 6:**
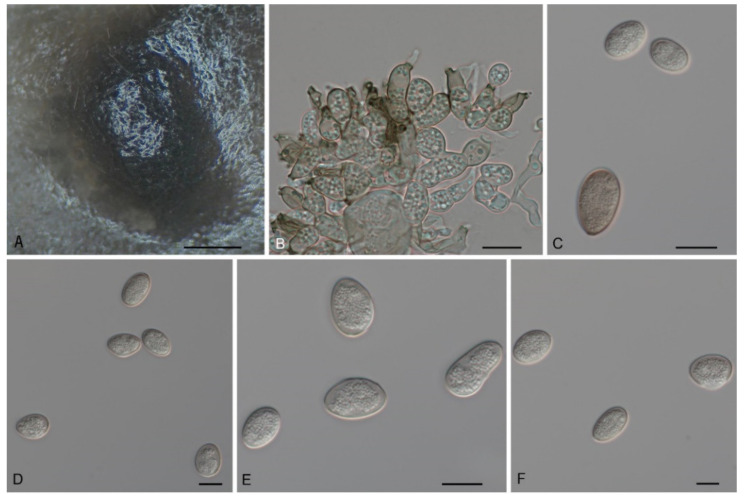
The morphology of *Tubakia americana* clade I (CFCC 54642). (**A**) Conidioma formed on PDA; (**B**) Conidiogenous cells giving rise to conidia; (**C**–**F**) Conidia. Scale bars: (**A**) = 100 μm; (**B**–**F**) = 10 μm.

**Figure 7:**
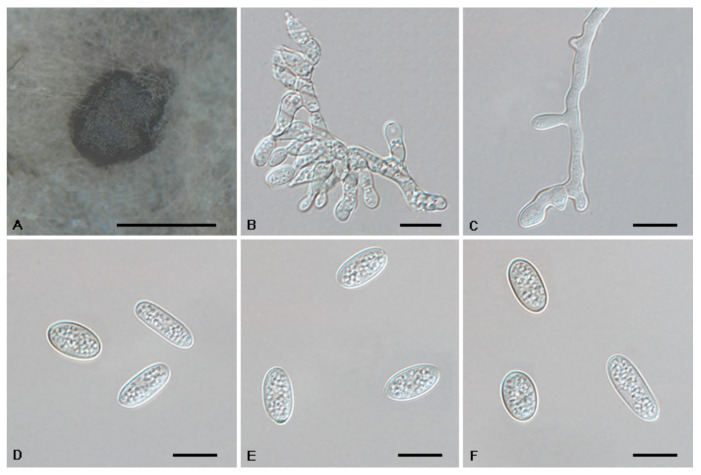
The morphology of *Tubakia americana* clade II (CFCC 56053). (**A**) Conidioma formed on PDA; (**B**–**C**) Conidiogenous cells giving rise to conidia; (**D**–**F**) Conidia. Scale bars: (**A**) = 300 μm; (**B**–**F**) = 10 μm.

*Tubakia americana* (Höhn.) T.C. Harr. & McNew, Antonie van Leeuwenhoek 111: 1016 (2018). Figure 6 and Figure 7.

Description: *Tubakia americana* clade I. Oak seed pathogen, also causing leaf spots or along necrotic leaf veins of *Quercus*. In vitro: Conidiomata sporodochial, usually globose or subglobose when viewed from above, formed on the agar surface; black, semi-submerged, 150–400 µm diam. Conidiophores were reduced to conidiogenous cells. Conidiogenous cells originating from sporodochia radiating, orientation outward, delicate, enlarged at the base and attenuated towards a narrow tip, cylindrical, rodlike to ampulliform, 11.5–15.1 × 4.7–9.5 µm. Conidia solitary, obovoid to ellipsoidal, 9.2–18.7 × 4.3–8.4(−11.5) µm, L/W = 1.1–3.0, aseptate, finely verrucoseto smooth, initially hyaline, later light brown, thick-walled, up to 2 μm wide. Microconidia were not observed. Sexual morph was not observed. *Tubakia americana* clade II. Causing leaf spots and seed spots. In vitro: Conidiomata sporodochial, usually globose or subglobose when viewed from above, formed on the agar surface; black, semi-submerged, 100–400 µm diam. Conidiophores were reduced to conidiogenous cells. Conidiogenous cells originating from sporodochia ampulliform or flask-shaped, smooth, hyaline, (6−)7.2–15.1(−16.7) × (1.5−)1.8–5.7(−6.8) μm. Conidia solitary, broad ellipsoid, ellipsoid-ovoid to short and broad subcylindrical, straight or slightly curved, both ends rounded or one end pointed, basal frills or truncated peg-like bases not observed, 10.0–13.3(−14.5) × (6.8−)7.7–12.9 μm, L/W = 1.0–1.7, finely verrucose to smooth, slightly lighter and wall thin when immature, up to 1 μm wide, slightly darker and wall thickened when ripening, up to 1.5 μm wide. Microconidia were not observed. Sexual morph was not observed.

Culture Characteristics: *Tubakia americana* clade I: Colonies on PDA incubated at 25 °C in the dark with an average radial growth rate of 9–10 mm/d and occupying an entire 90 mm Petri dish in 14 d, dark green on the bottom, aerial mycelium cottony, white initially, then becoming off-white. Colonies with optimal growth at 25 °C on MEA, attaining a diameter of 45–60 mm after 7 days, initially with a distinct ring of sparse aerial mycelium, later developing concentric rings of white to yellow aerial mycelium with wet conidial masses that are initially hyaline, becoming creamy white then faintly yellow, coalescing into large areas. *Tubakia americana* clade II: Colonies on PDA incubated at 25 °C in the dark with an average radial growth rate of 6–7 mm/d and occupying an entire 90 mm petri dish in 10 d. When young, yellow green mycelium mostly immersed; when old, in the middle dark green, with dark green parts covered with continuously growing white mycelia, dark green-to-black in reverse. Cultures incubated on MEA at 25 °C in darkness, attaining 29–33 mm diam. after 7 d (growth rate 4–5 mm diam./d), pale yellow to yellow with regular margin, white near the centre and hyphae immersion, reverse yellow to pale brown rings.

Material Examined: *Tubakia americana* clade I: CHINA, Henan Province, Xinyang City, Kikunshan National Nature Reserve, on leaf spots of *Quercus acutissima* and *Quercus aliena* var. *acuteserrata*, 20 September 2019, Dan-Ran Bian (living cultures CFCC 55115 and CFCC 55117); Shaanxi Province, Hanzhong City, Foping County, on leaf spots of *Quercus aliena* var. *acuteserrata* and *Quercus variabilis*, 10 September 2019, Dan-Ran Bian (living cultures CFCC 54642 and CFCC 54417); Anhui Province, Huangshan City, Mount Huang, on rotted seed of *Quercus glauca*, 16 September 2020, Cheng-Bin Wang (living cultures CFCC 55980, CFCC 55982 and CFCC 56051); Guangdong Province, Guangzhou City, on rotted seed of *Quercus glauca*, 20 September 2020, Cheng-Bin Wang (living culture CFCC 55975). *Tubakia americana* clade II: CHINA, Shaanxi Province, Xian City, Zhuque National Forest Park, on leaf spots of *Quercus aliena* var. *acuteserrata*, 25 August 2019, Dan-Ran Bian (living culture CFCC 54463); Shaanxi Province, Xian City, Zhuque Nationl Forest Park, on rotted seed of *Quercus aliena* var. *acuteserrata*, 16 September 2020, Ya-Quan Zhu (living cultures CFCC 55970, CFCC 55300 and CFCC 56053).

Host Range and Distribution: On *Quercus* (*acutissima*, *aliena* var. *acuteserrata*, *bicolor*, *coccinea*, *glauca*, *macrocarpa*, *robur*, *rubra*, *variabilis*), *Fagaceae*, China (Anhui Province, Guangdong Province, Henan Province and Shaanxi Province), North America (USA, Illinois, Iowa, Missouri, Wisconsin).

Notes: In this study, 12 isolates were obtained from diseased leaves of *Q. acutissima*, *Q. aliena* var. *acuteserrata* and *Q. variabilis*, as well as the rotted seeds of *Q*. *glauca*. These isolates were separated into two clades within the species *Tubakia americana* based on branch length (Figure 1).

**Figure 8:**
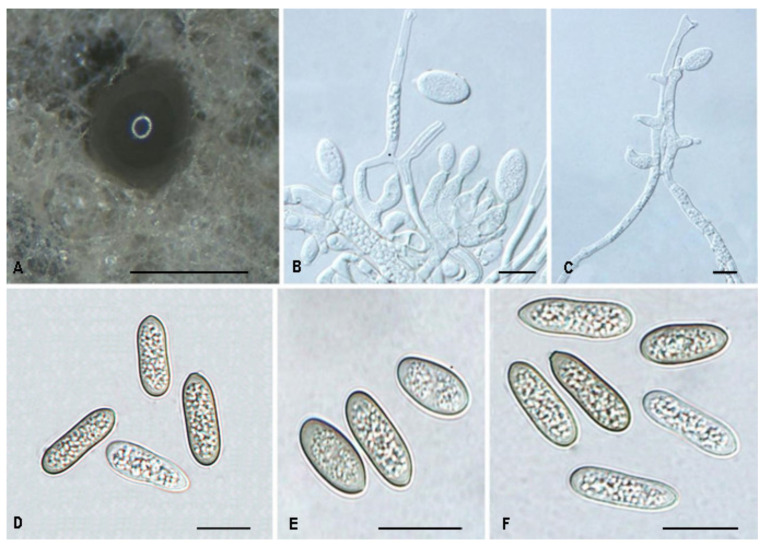
The morphology of *Tubakia cyclobalanopsidis* (CFCC 55979). (**A**) Conidiomata formed on PDA; (**B**–**C**) Conidiogenous cells giving rise to conidia; (**D**–**F**) Conidia. Scale bars: (**A**) = 300 μm; (**B**–**F**) = 10 μm.

*Tubakia cyclobalanopsidis* Ning Jiang & Y.Q. Zhu, sp. nov. Figure 8.

Mycobank no.: 845788

Diagnosis: *Tubakia cyclobalanopsidis* can be distinguished from its phylogenetically close species *T. paradryinoides* by smaller conidia.

Holotype: CHINA, Anhui Province, Huangshan City, Mount Huang, on rotted seeds of *Quercus glauca*, 16 September 2020, Cheng-Bin Wang (holotype CAF800064; ex-type culture, CFCC 55979).

Etymology: Named after the original genus name of the host *Quercus glauca, Cyclobalanopsis*.

Description: Causing a seed spot disease on *Q*. *glauca*, lesions subcircular to angular–irregular, 0.5–1 mm diam, brown to black, surrounded by a distinct margin, occasionally with a diffuse halo. In vitro: Conidiomata sporodochial, globose or subglobose, black, semi-submerged, 100–250 µm diam. Conidiophores reduced to conidiogenous cells. Conidiogenous cells originating from sporodochia ampulliform or flask-shaped, smooth, hyaline, 5.0–15.6 × 2.1–4.8 μm. Conidia aseptate, hyaline, smooth, globose to subglobose, occasionally broad ellipsoid-obovoid, cylindrical, 4.2–6(−6.5) × (2.6−)3–5.5 µm, L/W = 1.2–2.1, slightly lighter and wall thin when immature, up to 1 μm wide, slightly darker and wall thickened when ripening, up to 1.5 μm wide. Microconidia were not observed. Sexual morph was not observed.

Culture Characteristics: Colonies on PDA incubated at 25 °C in the dark with an average radial growth rate of 7–9 mm/d and reaching 52–57 mm diam. in 7 d. When young, round, dark green in the center and white at the edge, with some dark green parts covered with continuously growing mycelia. When old, tight, dark green and white at the edge, with dark green parts covered with continuously growing white mycelia. Cultures were incubated on MEA at 25 °C in darkness, attaining 37–41 mm diam. after 7 d (growth rate 5–6 mm diam./d), creamy white to faintly yellow with regular margin, white near the centre and hyphae clusters, reverse faintly yellow to yellow rings.

Material Examined: CHINA, Anhui Province, Huangshan City, Mount Huang, on rotted seeds of *Quercus glauca*, 16 September 2020, Cheng-Bin Wang (living cultures CFCC 55961 and CFCC 55973).

Host Range and Distribution: On *Quercus glauca*, *Fagaceae*, China (Anhui Province).

Notes: Three isolates from rot seeds of *Quercus glauca* were clustered into a well-supported clade here, newly described as *Tubakia cyclobalanopsidis*, which is distinct from any known species phylogenetically (Figure 1). Morphologically, *T. cyclobalanopsidis* can be distinguished by its phylogenetically close species *T. paradryinoides* by smaller conidia (4.2–6(−6.5) × (2.6−)3–5.5 μm in vitro in *T. cyclobalanopsidis* vs. 14–21 × 10–15 μm in vivo in *T. paradryinoides*) [1]. In addition, *T. cyclobalanopsidis* is separated from *T. paradryinoides* by 10 bp differences in ITS, 21 bp differences in *tef1* and 17 bp differences in *tub2*.

**Figure 9:**
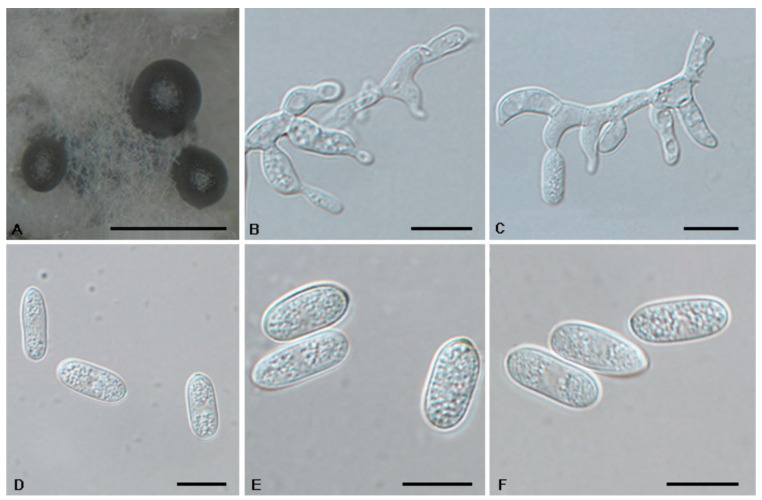
The morphology of *Tubakia dryinoides* (CFCC 54949). (**A**) Conidioma formed on PDA; (**B**–**C**) Conidiogenous cells giving rise to conidia; (**D**–**F**) Conidia. Scale bars: (**A**) = 500 μm; (**B**–**F**) = 10 μm.

*Tubakia dryinoides* C. Nakash., Fungal Systematics and Evolution 1: 80 (2018). Figure 9.

Description: Oak seeds rot, subcircular to angular–irregular, 0.5–1 mm diam, brown to black brown, margin indefinite or round, occasionally with a diffuse halo. In vitro: Conidiomata sporodochial, appeared within 10 days or longer, formed on agar surface, slimy, black, semi-submerged, 100–300 µm diam. Conidiophores reduced to conidiogenous cells. Conidiogenous cells originating from sporodochia hyaline, thin-walled, up to 1 μm wide, smooth, apex obtuse to truncate, radiating, cylindrical, conical, delicate, about (7.1−)9–13.5(−15.9) × (1.6−)2–3.6 µm. Conidia solitary, ellipsoid to obovoid, (8.7−)9.1–14.1 × 4.3–6.3 µm, L/W = 1.5–2.8, wall thin, up to 1 µm, at first subhyaline, later brownish, smooth, rod-like, apex pointed or round, base broadly rounded, with inconspicuous to conspicuous basal hilum (frill). Microconidia were not observed. Sexual morph was not observed.

Culture Characteristics: Colonies on PDA incubated at 25 °C in the dark with an average radial growth rate of 8–9 mm/d and occupying an entire 90 mm petri dish in 10 d; white initially, aerial mycelium cottony, then becoming moist yellow green, covered with grayish white mycelium. Cultures incubated on MEA at 25 °C in darkness, attaining 61–74 mm diam after 10 days, margin scalloped, creamy white initially, then with fluffy pale brown to dark brown mycelia, yellow, pale brown to brown in reverse, with a cream white edge.

Material Examined: CHINA, Anhui Province, Hefei City, Shushan Forest Park, on leaf spots of *Quercus acutissima* and *Quercus aliena* var. *acuteserrata*, 1 Spetember 2019, Dan-Ran Bian (living cultures CFCC 54949 and CFCC 54975); Anhui Province, Huangshan City, Mount Huang, on rotted seed of *Quercus glauca*, 16 September 2020, Cheng-Bin Wang (living cultures CFCC 55958, CFCC 55983 and CFCC55966).

Host Range and Distribution: On *Castanea crenata* and *Quercus* (*acutissima*, *aliena* var. *acuteserrata*, *glauca*, *phillyraeoides*), *Fagaceae*, China (Anhui Province) and Japan.

Notes: The holotype of *T. dryinoides* was collected from leaves of *Q. phillyraeoides*. In this study, isolates collected from diseased leaves of *Q. acutissima*, *Q. aliena* var. *acuteserrata* and seeds of *Q. glauca*, which formed a well-supported clade with the ex-type strain MUCC2292 (Figure 1).

**Figure 10:**
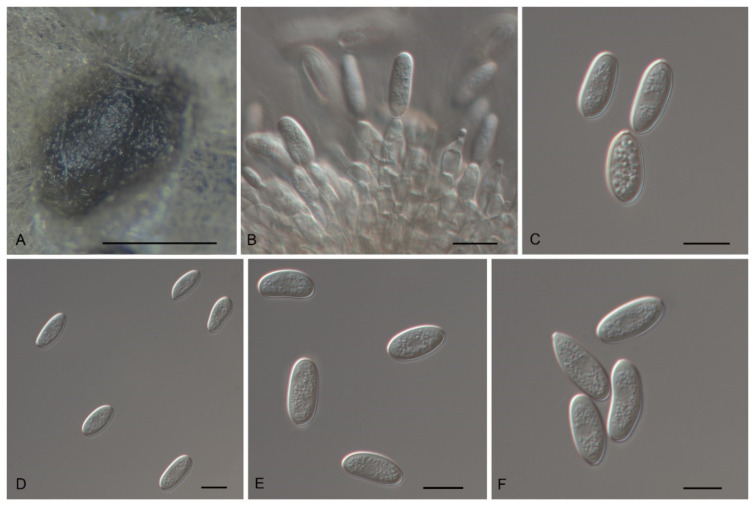
The morphology of *Tubakia koreana* (CFCC 54968). (**A**) Conidioma formed on PDA; (**B**) Conidiogenous cells giving rise to conidia; (**C**–**F**) Conidia. Scale bars: (**A**) = 100 μm; (**B**–**F**) = 10 μm.

*Tubakia koreana* H.Y. Yun, Mycotaxon 135(1): 225 (2020). Figure 10.

Description: Causing leaf blight on leaves, first symptom visible as small pale-brownish, lesions later expanding along the veins and blades. In vitro: Conidiomata sporodochial, appeared within 10 days or longer, formed on agar surface, slimy, black, semi-submerged, acute and cornuted at the margin that was fringed and unattached to the substrate, 150–400 µm diam. Conidiophores reduced to conidiogenous cells. Conidiogenous cells originating from sporodochia subcylindrical, subclavate, partly attenuated towards the tip, 11.5–16.3 × 5.6–7.5 µm, hyaline, thin-walled, up to 1 μm wide, smooth. Conidia solitary, ellipsoid-obovoid, fusiform, oblong, straight to slightly curved, (9−)10.1–19.0(−22.3) × 4.3–10.3 µm, L/W = 1.1–3.5, wall thin, up to 1 µm wide, at first subhyaline, later pale olivaceous to brownish. Microconidia were not observed. Sexual morph was not observed.

Culture Characteristics: Colonies on PDA incubated at 25 °C in the dark with an average radial growth rate of 7–8 mm/d and reaching 54–59 mm diam. in 7 d, aerial mycelium cottony, creamy white initially, then becoming pale yellow. Cultures incubated on MEA at 25 °C in darkness, attaining 55–60 mm diam. after 7 d (growth rate 7–9 mm diam./d), margin scalloped, at first creamy white, forming concentric rings of aerial hyphae, reverse in the middle yellow, yellow to white brown towards the rim.

Material Examined: CHINA, Henan Province, Xinyang City, Kikunshan National Nature Reserve, on leaf spots of *Quercus acutissima*, 20 September 2019, Dan-Ran Bian (living culture CFCC 54629); Anhui Province, Huangshan City, Mount Huang, on rotted seed of *Quercus glauca*, 16 September 2020, Cheng-Bin Wang (living cultures CFCC 55990, CFCC 55976, CFCC 56113, CFCC 55967, CFCC 55977, CFCC 55963, CFCC 55989 and CFCC 55988); Anhui Province, Hefei City, Shushan Forest Park, on leaf spots of *Quercus acutissima*, 1 September 2019, Dan-Ran Bian (living culture CFCC 54968); Henan Province, Pingdingshan City, Yaoshan Mountain, on leaf spots of *Quercus variabilis*, 18 September 2019, Dan-Ran Bian (living cultures CFCC 54477 and CFCC 54488); Anhui Province, Hefei City, Zipeng Mountain, on leaf spots of *Castanea mollissima*, 4 September 2019, Dan-Ran Bian (living culture CFCC 54916).Host range and distribution—on *Castanea mollissima* and *Quercus* (*acutissima*, *alienoserratoides*, *glauca*, *mongolica*, *serrata*, *variabilis*), *Fagaceae*, China (Anhui Province and Henan Province) and Korea.

Notes: *T. koreana* was proposed based on morphological and ITS sequence data [8]. The holotype of *T. koreana* was collected from *Q. mongolica* in Korea. In the present study, 13 isolates collected from diseased leaves of *C*. *mollissima*, *Q. acutissima*, *Q. variabilis*, and rotten seeds of *Q. glauca*, which were the same as the ex-type stain KCTC 46072 in the ITS sequence. The conidia size of our isolates is similar to the original description of *T. koreana* [8]. In addition, we sequenced *tef1* and *tub2* sequences of this species.

**Figure 11:**
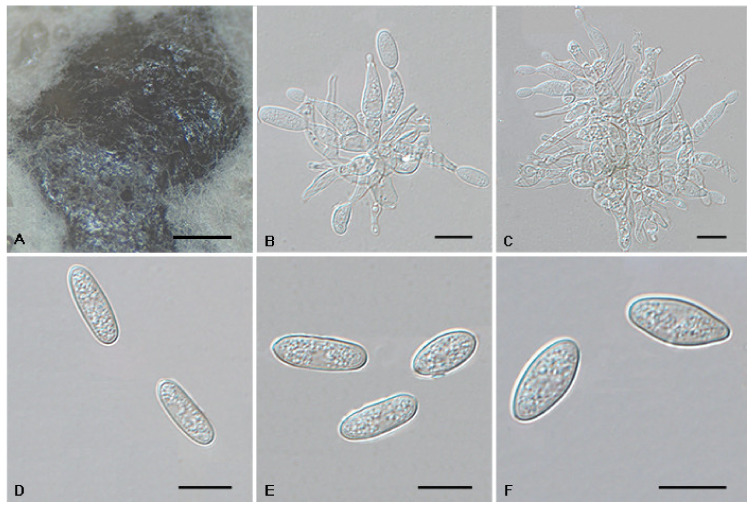
The morphology of *Tubakia paradryinoides* (CFCC 55984). (**A**) Conidiomata formed on PDA; (**B**–**C**) Conidiogenous cells giving rise to conidia; (**D**–**F**) Conidia. Scale bars: (**A**) = 100 μm; (**B**–**F**) = 10 μm.

*Tubakia paradryinoides* C. Nakash., Fungal Systematics and Evolution 1: 80 (2018). Figure 11.

Description: Oak seeds rot, forming crustose conidiomata on the surface of leaves and causing leaf spots. In vitro: Conidiomata sporodochial, appeared within 10 days or longer, formed on the agar surface, slimy, black, semi-submerged, 200–750 µm diam. Conidiophores reduced to conidiogenous cells. Conidiogenous cells originating from sporodochia conical to ampulliform, (7.7−)8.1–17.5 × 2–4.6(−5.8) µm, subhyaline to pale brown, thin-walled, up to 1 μm wide, smooth, apex obtuse to truncate, conidiogenesis phialidic, sometimes forming indistinct periclinal thickenings. Conidia solitary, broad ellipsoid-obovoid, (10.4−)11.4–16.1(−19.2) × (2.6−)3–5.1(−5.5) µm, L/W = 2.5–4.8, wall thin, up to 1 µm wide, hyaline to subhyaline, apex pointed and rounded, base broadly rounded, with inconspicuous to conspicuous basal hilum. Microconidia were not observed. Sexual morph was not observed.

Culture Characteristics: Colonies on PDA at 25 °C for 10 days attain 78–90 mm in diameter. When young, round, cream white in the center, with some moist mycelium; when old, hyphae lush, gray to ash black, white at the edge. On MEA with optimal growth at 25 °C, attaining 43–50 mm after 7 days, margin scalloped, faintly yellow in the centre and with a cream white edge, they have wrinkles, yellow to brown in reverse, white at the edge. 

Material Examined: CHINA, Anhui Province, Huangshan City, Mount Huang, on rotted seed of *Quercus glauca*, 16 September 2020, Cheng-Bin Wang (living cultures CFCC 55984, CFCC 55959, CFCC 55974 and CFCC 55972).

Host range and distribution: on *Quercus* (*acutissima* and *glauca*), *Fagaceae*, China (Anhui Province) and Japan.

Notes: The holotype of *T. paradryinoides* was collected from *Q. acutissima* [1]. In the present study, isolates were collected from the diseased seeds of *Q. glauca.* Four isolates clustered in the *T. paradryinoides* clade by strong support (Figure 1). *T. paradryinoides* is phylogenetically close to *T. cyclobalanopsidis*. However, *T. paradryinoides* can be distinguished from *T. cyclobalanopsidis* by larger conidia (14–21 × 10–15 µm in *T. paradryinoides* vs. 4.2–6(−6.5) × (2.6−)3–5.5 µm in *T. cyclobalanopsidis*) [1].

**Figure 12:**
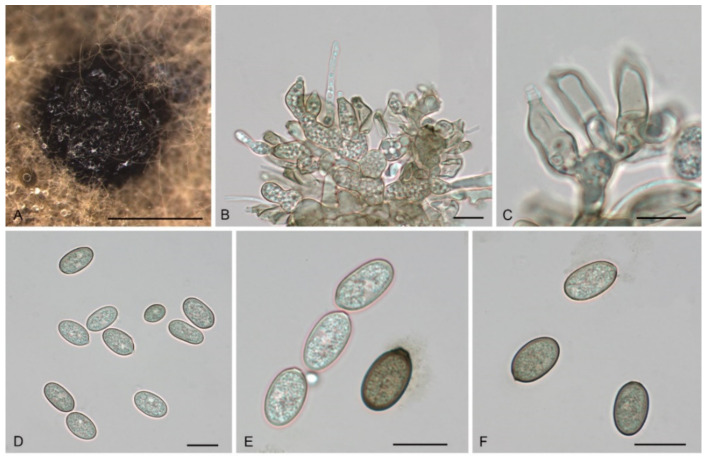
The morphology of *Tubakia quercicola* (CFCC 55106). (**A**) Conidiomata formed on PDA; (**B**–**C**) Conidiogenous cells giving rise to conidia; (**D**–**F**) Conidia. Scale bars: (**A**) = 100 μm; (**B**–**F**) = 10 μm.

*Tubakia quercicola* Ning Jiang & Y.Q. Zhu, sp. nov. Figure 12.

Mycobank no.: 845789

Diagnosis: *Tubakia quercicola* can be distinguished from its phylogenetically close species *T*. *dryina* by sizable differences in its ITS, *tef1*, and *tub2* sequences.

Holotype: CHINA, Shaanxi Province, Baoji City, Panjiawan Forest Park, on leaf spots of *Quercus aliena* var. *acuteserrata*, 27 August 2019, Dan-Ran Bian (holotype CAF800065; ex-type culture, CFCC 55106); *Ibid*. (living cultures CFCC 54426 and CFCC 54754).

Etymology: Referring to the host genus *Quercus*.

Description: Causing leaf spots, subcircular to angular-irregular, greyish white, margin distinct, brown to fuscous. In vitro: Conidiomata sporodochial, appeared within 7 days or longer, formed on the agar surface, slimy, black, semi-submerged, 100–250 µm diam. Conidiophores originating from sporodochia indistinct, often reduced to conidiogenous cells. Conidiogenous cells hyaline, smooth, multiguttulate, cylindrical to ampulliform, attenuate towards apex, phialidic, 10.9–19.3 × 4.2–6.9 µm. Conidia were (8.3−) 11.8–17.6 (−18.2) × 6.0–8.9 (−10.6) µm, length/width ratio 1.4–2.6, blastic, subglobose, broad ellipsoid to ellipsoid, hyaline, becoming pale yellowish brown, walls smooth, becoming thicker with age, base rounded or with truncate basal hilum. Microconidia were not observed. Sexual morph was not observed.

Culture Characteristics: Colonies on PDA were incubated at 25 °C in the dark with an average radial growth rate of 9–11 mm/d and occupying an entire 90 mm Petri dish in 14 d, dark green on the bottom, aerial mycelium cottony, white initially, then becoming greyish. Optimal growth at 25 °C on MEA in darkness, colonies attaining 39–45 mm after 7 days, dingy white to pale yellow with regular margin, becoming yellowish gray with concentric rings in reverse, conidial formation not observed. The colony growth rate on MEA reached 6 mm/day, which is a growth that is slower than on PDA.

Material Examined: CHINA, Guizhou Province, Zunyi City, Kuankuoshui National Nature Reserve, on rotted seed of *Quercus aliena* var. *acuteserrata*, 20 August 2019, Dan-Ran Bian (living culture CFCC 54312); Shaanxi Province, Xian City, Zhuque National Forest Park, on leaf spots of *Quercus aliena* var. *acuteserrata*, 25 August 2019, Dan-Ran Bian (living cultures CFCC 54471, CFCC 54284, CFCC 54326, CFCC 54912 and CFCC 54294).

Host range and distribution: on *Quercus aliena* var. *acuteserrata*, *Fagaceae*, China (Guizhou Province and Shaanxi Province).

Notes: The phylogenetic analysis of a combined three genes alignment (ITS, *tef1* and *tub2*) showed that *T*. *quercicola* clustered into a well-supported clade. Morphologically, *T*. *quercicola* is similar to *T*. *dryina* in conidial size [1]. However, *T*. *quercicola* can be distinguished from *T*. *dryina* by sequence data (5/631 in ITS; 25/604 in *tef1* and 20/535 in *tub2*). Furthermore, the MEA’s colony colour of *T*. *quercicola* is different from *T*. *dryina* (surface: creamy white to faintly yellow vs. creamy white, dark grey, yellow to medium brown) [1].

### 3.5. Pathogenicity Test

The results of the pathogenicity test on four hosts are shown in Table 4. We can see that the aggressiveness of the tested species for different leaves differed significantly. For instance, *T*. *paradryinoides* was not pathogenic to *Q*. *aliena* var. *acutiserrata*, but the other fungal species could cause lesions on all tested host leaves. In addition, *T*. *paradryinoides* had a high infection rate in *C*. *mollissima*, *Q. acutissima* and *Q*. *variabilis*, but was not pathogenic to *Q*. *aliena* var. *acutiserrata*.

## 4. Discussion

It is well known that *Tubakia* species have a wide geographic distribution mainly inhabiting *Fagaceae* hosts. *Tubakia* may appear on leaf or twig tissues simultaneously with other agents. Species of *Tubakia* may have an endophytic phase of growth [19,33,34,35]. Some endophytic *Tubakia* species have mutualistic interactions with their plant hosts, including the concept of a sentinel tree [36]. Currently, the majority of *Tubakia* species are mainly endophytes. However, *T*. *iowensis* cause a serious leaf disease (bur oak blight), and *T*. *hallii* and *T*. *macnabbii* have also been related to the significant defoliation of *Quercus* spp. [11,12].

In this study, we investigated the diversity of pathogens in *Fagaceae* plants in China and obtained 46 isolates belonging to *Tubakia*. Based on morphology and a concatenated three-gene phylogenetic analysis, the isolates were assigned to six species (viz. *T*. *americana*, *T*. *cyclobalanopsidis*, *T*. *dryinoides*, *T*. *koreana*, *T*. *paradryinoides* and *T*. *quercicola*). Each species formed a well-supported monophyletic clade in the phylogenetic analysis. Since the inception of *Tubakia* in 1913, its delimitation has undergone several changes. Twenty-one species are phylogenetically recognized in *Tubakia*, including *T. cyclobalanopsidis* and *T*. *quercicola* spp. nov. from the present study [1,5,8,9].

*Tubakia cyclobalanopsidis* sp. nov. appeared to be a sister species of *T*. *paradryinoides* in the phylogram (Figure 1), but differed from *T*. *paradryinoides* by 10, 21 and 17 variable nucleotide sites in the ITS, *tef1* and *tub2* genes, respectively. Morphologically, *T*. *cyclobalanopsidis* differs from *T*. *paradryinoides* by producing smaller conidia. *Tubakia quercicola*, newly discovered in this study, is a remarkable species in *Tubakia*. Morphologically, *T*. *quercicola* can be easily identified as a member of *T*. *koreana* or *T*. *melnikiana*, due to the size of conidiogenous cells and conidia. However, phylogenetic analyses demonstrated that it is a new clade (Figure 1). Bayesian inference and maximum likelihood analyses showed that protein-coding genes (*tef1* and *tub2*), mostly *tef1*, have sufficient discriminatory power to differentiate *T*. *quercicola.*

The results revealed two clades within *T*. *americana*. The independence of *T*. *americana* clade I as a distinct clade is mainly supported by its unique *tef1* sequence, which influences its position in the phylogenetic tree. Morphologically, all isolates in the two clades share the typical characteristics of *T. americana* [1,3]. *T*. *dryinoides* was firstly discovered on *Q*. *acutissima*, *Q*. *aliena* var. *acutiserrata* and *Q*. *glauca*. *T*. *koreana* was firstly described on *C*. *mollissima*, *Q*. *glauca* and *Q*. *variabilis. T*. *paradryinoides* was firstly described on *Q*. *glauca*. Therefore, this study expands the habitat and host of *T*. *americana*, *T*. *dryinoides*, *T*. *koreana* and *T*. *paradryinoides* in China.

Pathogenicity tests of the six species identified in the present study were conducted on four host plants, which shows that *T*. *quercicola* had the highest incidence (Table 4). Furthermore, all tested *Tubakia* species showed significantly different lesion diameters on leaves (Table 4). Therefore, our studies revealed a broad diversity in pathogenicity and aggressiveness among six *Tubakia* species.

The proper identification of fungal species is necessary in disease control [37,38,39]. Our knowledge of fungi and their relationships with plant hosts has increased exponentially due to the progress in bioinformatics and molecular phylogenetics. *Tubakia* species are endophytes in leaves and twigs of many species, but can also cause conspicuous leaf symptoms as plant pathogens. Therefore, identification of *Tubakia* species associated with hosts, as well as their lifestyles, is important. This study conducted a large-scale investigation of *Tubakia* associated with *Fagaceae* in China and provides morphological, molecular, and biological characterizations of these *Tubakia* species. This study not only enhances our understanding of the diversity of *Tubakia* species associated with *Fagaceae*, but also enriches knowledge of the host diversity of *Tubakia* species.

## 5. Conclusions

Six *Tubakia* species are identified from fagaceous hosts in China based on morphology and phylogeny; viz. *T. americana*, *T. cyclobalanopsidis*, *T. dryinoides*, *T*. *koreana*, *T*. *paradryinoides* and *T*. *quercicola*. This study enriches the species diversity of the genus, which will also promote its taxonomy and phylogeny.

## Figures and Tables

**Table 1:** Loci used in this study with PCR primers, and the process.

Loci	Primers	PCR: Thermal Cycles: (Annealing Temp. in Bold)	Reference
ITS	ITS/ITS4	(95 °C:30 s, **48 °C**:30 s, 72 °C:1 min) × 35 cycles	[23]
*tef1*	EF1–728F/EF1–986R	(95 °C:15 s, **54 °C**:20 s, 72 °C:1 min) × 35 cycles	[24]
*tub2*	T1(Bt2a)/Bt2b	(95 °C:30 s, **55 °C**:30 s, 72 °C:1 min) × 35 cycles	[25,26]

**Table 2:** Strains and GenBank accession numbers used in this study.

Species	Country	Host	Strain	GenBank Accession Number		
				ITS	*tef1*	*tub2*
***Involutiscutellula rubra***	**Japan**	***Q. phillyraeoides***	CBS 192.71 *	MG591899	MG592086	MG592180
*I. rubra*	Japan	*Q. phillyraeoides*	MUCC2303	MG591900	MG592087	MG592181
*I. rubra*	Japan	*Q. phillyraeoides*	MUCC2305	MG591902	MG592089	MG592182
*Melanconis groenlandica*	Greenland	*Betula nana*	CBS 116540 *	KU878552	KU878554	KU878555
*Oblongisporothyrium castanopsidis*	Japan	*Castanea cuspidata*	CBS 124732 *	MG591849	MG592037	MG592131
*Paratubakia subglobosa*	Japan	*Q. glauca*	CBS 124733 *	MG591913	MG592102	MG592194
*P. subglobosoides*	Japan	*Q. glauca*	MUCC2293 *	MG591915	MG592104	MG592196
*Racheliella wingfieldiana*	South Africa	*Syzigium guineense*	CBS 143669	MG591911	MG592100	MG592192
*Sphaerosporithyrium mexicanum*	Mexico	*Q. eduardi*	CPC 31361	MG591894	MG592081	MG592175
*S. mexicanum*	Mexico	*Q. eduardi*	CPC 32258	MG591895	MG592082	MG592176
*S. mexicanum*	Mexico	*Q. eduardi*	CPC 33021	MG591896	MG592083	MG592177
*Tubakia americana*	USA	*Q.macrocarpa*	CBS 129014	MG591873	MG592058	MG592152
***T. americana***	**China**	***Q. acutissima***	**CFCC 55115**	**OP114595**	**OP254203**	**OP254249**
***T. americana***	**China**	***Q. aliena* var. *acuteserrata***	**CFCC 54642**	**OP114596**	**OP254204**	**OP254250**
***T. americana***	**China**	***Q. aliena* var. *acuteserrata***	**CFCC 55117**	**OP114597**	**OP254205**	**OP254251**
***T. americana***	**China**	***Q. glauca***	**CFCC 55980**	**OP114598**	**OP254206**	**OP254252**
***T. americana***	**China**	***Q. glauca***	**CFCC 55982**	**OP114599**	**OP254207**	**OP254253**
***T. americana***	**China**	***Q. variabilis***	**CFCC 54417**	**OP114600**	**OP254208**	**OP254254**
***T. americana***	**China**	***Q. glauca***	**CFCC 55975**	**OP114601**	**OP254209**	**OP254255**
***T. americana***	**China**	***Q. glauca***	**CFCC 56051**	**OP114602**	**OP254210**	**OP254256**
***T. americana***	**China**	***Q. aliena* var. *acuteserrata***	**CFCC 55970**	**OP114603**	**OP254211**	**OP254257**
***T. americana***	**China**	***Q. aliena* var. *acuteserrata***	**CFCC 54463**	**OP114604**	**OP254212**	**OP254258**
***T. americana***	**China**	***Q. aliena* var. *acuteserrata***	**CFCC 55300**	**OP114605**	**OP254213**	**OP254259**
***T. americana***	**China**	***Q. aliena* var. *acuteserrata***	**CFCC 56053**	**OP114606**	**OP254214**	**OP254260**
*T. californica*	USA	*Q. agrifolia*	CPC 31496	MG591829	MG592017	MG592111
*T. californica*	USA	*Q. wislizeni*	CPC 31499	MG591832	MG592020	MG592114
*T. californica*	USA	*Q. kelloggii*	CPC 31504	MG591834	MG592022	MG592116
*T. californica*	USA	*Lithocarpus densiflorus*	CPC 31514	MG591843	MG592031	MG592125
*T. californica*	USA	*Chrysolepis chrysophylla*	CPC 31517	MG591846	MG592034	MG592128
*T. californica*	Mexico	*Q. canbyi*	CPC 32250	MG591847	MG592035	MG592129
***T. cyclobalanopsidis***	**China**	***Q. glauca***	**CFCC 55961**	**OP114638**	**OP254246**	**OP329289**
***T. cyclobalanopsidis***	**China**	***Q. glauca***	CFCC 55979 *	**OP114639**	**OP254247**	**OP329290**
***T. cyclobalanopsidis***	**China**	***Q. glauca***	**CFCC 55973**	**OP114640**	**OP254248**	**OP329291**
*T. dryina*	Italy	*Q. robur*	CBS 112097 *	MG591851	MG592039	MG592133
*T. dryina*	Netherlands	*Quercus* sp.	CBS 114912	MG591853	MG592041	MG592135
*T. dryina*	USA	*Q. alba*	CBS 129016	MG591870	MG592056	MG592150
*T. dryina*	USA	*Q. macrocarpa*	CBS 129018	MG591871	MG592057	MG592151
*T. dryinoides*	France	*Quercus* sp.	CBS 329.75	MG591874	MG592059	MG592153
*T. dryinoides*	Japan	*Q. phillyraeoides*	MUCC2292 *	MG591878	MG592063	MG592157
***T. dryinoides***	**China**	***Q. glauca***	**CFCC 55958**	**OP114607**	**OP254215**	**OP254261**
***T. dryinoides***	**China**	***Q. glauca***	**CFCC 55983**	**OP114608**	**OP254216**	**OP254262**
***T. dryinoides***	**China**	***Q. glauca***	**CFCC 55966**	**OP114609**	**OP254217**	**OP254263**
***T. dryinoides***	**China**	***Q. acutissima***	**CFCC 54949**	**OP114610**	**OP254218**	**OP254264**
***T. dryinoides***	**China**	***Q. aliena* var. *acuteserrata***	**CFCC 54975**	**OP114611**	**OP254219**	**OP254265**
*T. hallii*	USA	*Q. stellata*	CBS 129013 *	MG591880	MG592065	MG592159
*T. hallii*	USA	*Q. stellata*	CBS 129015	MG591881	MG592066	MG592160
*T. hallii*	Iran	*Quercus* sp.	CPC 23753	MG591884	MG592069	MG592163
*T. iowensis*	USA	*Q. macrocarpa*	CBS 129012 *	MG591879	MG592064	MG592158
*T. iowensis*	USA	*Q. macrocarpa*	CBS 129017	MG591882	MG592067	MG592161
*T. iowensis*	USA	*Q. macrocarpa*	CBS 129019	MG591883	MG592068	MG592162
*T. japonica*	Japan	*C. crenata*	CBS 191.71	MG591885	MG592070	MG592164
*T. japonica*	Japan	*C. crenata*	MUCC2300	MG591887	MG592074	MG592168
***T. koreana***	**China**	***Q. glauca***	**CFCC 55990**	**OP114616**	**OP254224**	**OP254270**
***T. koreana***	**China**	***Q. glauca***	**CFCC 55976**	**OP114617**	**OP254225**	**OP254271**
***T. koreana***	**China**	***Q. glauca***	**CFCC 56113**	**OP114618**	**OP254226**	**OP254272**
***T. koreana***	**China**	***Q. acutissima***	**CFCC 54629**	**OP114619**	**OP254227**	**OP254273**
***T. koreana***	**China**	***Q. glauca***	**CFCC 55967**	**OP114620**	**OP254228**	**OP254274**
***T. koreana***	**China**	***Q. glauca***	**CFCC 55977**	**OP114621**	**OP254229**	**OP254275**
***T. koreana***	**China**	***Q. glauca***	**CFCC 55963**	**OP114622**	**OP254230**	**OP254276**
***T. koreana***	**China**	***Q. acutissima***	**CFCC 54968**	**OP114623**	**OP254231**	**OP254277**
***T. koreana***	**China**	***Q. variabilis***	**CFCC 54488**	**OP114624**	**OP254232**	**OP254278**
***T. koreana***	**China**	***Q. variabilis***	**CFCC 54477**	**OP114625**	**OP254233**	**OP254279**
***T. koreana***	**China**	***Q. glauca***	**CFCC 55989**	**OP114626**	**OP254234**	**OP254280**
***T. koreana***	**China**	***Q. glauca***	**CFCC 55988**	**OP114627**	**OP254235**	**OP254281**
***T. koreana***	**China**	***C. mollissima***	**CFCC 54916**	**OP114628**	**OP254236**	**OP254282**
*T. liquidambaris*	USA	*Liquidambar styraciflua*	CBS 139744	MG605068	MG603578	NA
*T. lushanensis*	China	*Q. palustris*	SAUCC 1921	MW784677	MW842262	MW842265
*T. lushanensis*	China	*Q. palustris*	SAUCC 1923 *	MW784678	MW842261	MW842264
*T. macnabbii*	USA	*Q. palustris*	CBS 137349 *	MG605069	MG603579	NA
*T. melnikiana*	Mexico	*Q. canbyi*	CPC 32249	MG591889	MG592076	MG592170
*T. melnikiana*	Mexico	*Q. laeta*	CPC 32254	MG591892	MG592079	MG592173
*T. melnikiana*	Mexico	*Q. canbyi*	CPC 32255 *	MG591893	MG592080	MG592174
*T. oblongispora*	Japan	*Q. serrata*	MUCC2295 *	MG591897	MG592084	MG592178
*T. paradryinoides*	Japan	*Q. acutissima*	MUCC2294 *	MG591898	MG592085	MG592179
***T. paradryinoides***	**China**	***Q. glauca***	**CFCC 55984**	**OP114612**	**OP254220**	**OP254266**
***T. paradryinoides***	**China**	***Q. glauca***	**CFCC 55959**	**OP114613**	**OP254221**	**OP254267**
***T. paradryinoides***	**China**	***Q. glauca***	**CFCC 55974**	**OP114614**	**OP254222**	**OP254268**
***T. paradryinoides***	**China**	***Q. glauca***	**CFCC 55972**	**OP114615**	**OP254223**	**OP254269**
***T. quercicola***	**China**	***Q. aliena* var. *acuteserrata***	**CFCC 54426**	**OP114629**	**OP254237**	**OP254283**
***T. quercicola***	**China**	***Q. aliena* var. *acuteserrata***	**CFCC 54471**	**OP114630**	**OP254238**	**OP254284**
***T. quercicola***	**China**	***Q. aliena* var. *acuteserrata***	**CFCC 54284**	**OP114631**	**OP254239**	**OP254285**
***T. quercicola***	**China**	***Q. aliena* var. *acuteserrata***	**CFCC 54326**	**OP114632**	**OP254240**	**OP254286**
***T. quercicola***	**China**	***Q. aliena* var. *acuteserrata***	**CFCC 54312**	**OP114633**	**OP254241**	**OP254287**
***T. quercicola***	**China**	***Q. aliena* var. *acuteserrata***	**CFCC 54754**	**OP114634**	**OP254242**	**OP254288**
***T. quercicola***	**China**	***Q. aliena* var. *acuteserrata***	CFCC 55106 *	**OP114635**	**OP254243**	**OP254289**
***T. quercicola***	**China**	***Q. aliena* var. *acuteserrata***	**CFCC 54912**	**OP114636**	**OP254244**	**OP254290**
***T. quercicola***	**China**	***Q. aliena* var. *acuteserrata***	**CFCC 54294**	**OP114637**	**OP254245**	**OP254291**
*T. seoraksanensis*	South Korea	*Q. mongolica*	CBS 127490 *	MG591907	MG592094	MG592186
*T. seoraksanensis*	China	*Q. mongolica*	CPC 26552	MG591909	MG592097	MG592189
*T. sierrafriensis*	Mexico	*Q. eduardi*	CPC 33020 *	MG591910	MG592099	MG592191
*T. suttoniana*	Netherlands	*Quercus* sp.	CBS 114911	MG591916	MG592105	MG592197
*T. suttoniana*	Netherlands	*Q. robur*	CBS 115006	MG591917	MG592106	MG592198
*T. suttoniana*	New Zealand	*Q. cerris*	CBS 229.77	MG591919	MG592108	MG592200
*T. tiffanyae*	USA	*Q. rubra*	CBS 137345 *	MG605081	MG603581	NA

Note: NA, not applicable. Ex-type strains are marked with *, and strains from present study are in bold.

**Table 3:** An overview of colony diameters at various temperatures.

Species	Media	Lesion Diameter ^1^ (cm)							
		5 °C	10 °C	15 °C	20 °C	25 °C	30 °C	35 °C	40 °C
*Tubakia americana* clade I	PDA	0	1.7 ± 0.1	2.7 ± 0.1	4.8 ± 0.2	6.5 ± 0.2	6 ± 0.3	2.7 ± 0.1	0
	MEA	0	0	1.4 ± 0.1	3 ± 0.1	4.7 ± 0.1	4.6 ± 0.2	2.2 ± 0.1	0
*Tubakia americana* clade II	PDA	0	0	2.4 ± 0.1	4.4 ± 0.2	6.8 ± 0.1	2.8 ± 0.1	0	0
	MEA	0	0	0.7	2.2 ± 0.1	3.2 ± 0.1	1.2 ± 0.1	0	0
*Tubakia cyclobalanopsidis*	PDA	0.7	1.4 ± 0.1	2.4 ± 0.1	4.6 ± 0.1	5.4 ± 0.1	5.8 ± 0.2	2.5 ± 0.1	0
	MEA	0	1	1.6 ± 0.1	2.5	3.8 ± 0.1	4.9 ± 0.2	2.4	0
*Tubakia dryinoides*	PDA	0	1.6 ± 0.1	2.5 ± 0.1	4.8 ± 0.1	6.1 ± 0.2	6.9 ± 0.3	4.2 ± 0.1	0
	MEA	0	0	1.6 ± 0.1	3.8 ± 0.1	5 ± 0.1	5.1 ± 0.2	3.6 ± 0.1	0
*Tubakia koreana*	PDA	1.2	2.5 ± 0.1	4.1 ± 0.1	5.4 ± 0.2	5.6 ± 0.1	4.5 ± 0.1	2 ± 0.1	0
	MEA	0	1.6 ± 0.1	2.6 ± 0.1	4.1 ± 0.1	5.6 ± 0.2	5.8 ± 0.2	2.1	0
*Tubakia paradryinoides*	PDA	0	1.9 ± 0.1	3.6 ± 0.1	5.8 ± 0.2	6.1 ± 0.2	7.6 ± 0.2	4 ± 0.1	0
	MEA	0	1.6 ± 0.1	2.4 ± 0.1	3 ± 0.1	4.5 ± 0.1	5.1 ± 0.1	3.1 ± 0.1	0
*Tubakia quercicola*	PDA	0	2.2 ± 0.1	2.6 ± 0.1	5 ± 0.1	7.3 ± 0.3	3.6 ± 0.1	1.1	0
	MEA	0	0	1.4 ± 0.1	2.7 ± 0.1	4.2 ± 0.1	3 ± 0.1	1	0

^1^ Lesion Diameter: mean ± standard error.

**Table 4:** Pathogenicity testing for six *Tubakia* species.

Species	Days	Lesion Diameter (mm)				Incidence Rate (%)				Total
		*Castanea mollissima*	*Quercus acutissima*	*Q. variabilis*	*Q. aliena var.acuteserrata*	*C. mollissima*	*Q. acutissima*	*Q. variabilis*	*Q. aliena var.acuteserrata*	
*Tubakia americana* clade I	7	0–15	0–8	0–6	7–8	66.7%	66.7%	83.3%	100%	79.18%
	14	0–23	0–11	5–19	2–26	66.7%	66.7%	83.3%	100%	79.18%
*Tubakia americana* clade II	7	0–5	0–2	0–2	9–11	50%	33.3%	33.3%	100%	54.15%
	14	0–9	0–5	0–11	22–29	83.3%	83.3%	83.3%	100%	87.48%
*Tubakia cyclobalanopsidis*	7	3–22	0–5	0–1	6–9	100%	66.7%	83.3%	100%	87.5%
	14	4–25	0–7	0–1	6–11	100%	66.7%	83.3%	100%	87.5%
*Tubakia dryinoides*	7	0–11	5–8	4–12	3–6	83.3%	100%	100%	100%	95.83%
	14	0–23	8–15	5–2	6–16	83.3%	100%	100%	100%	95.83%
*Tubakia koreana*	7	0.2–15	2–18	5–10	0–10	100%	100%	100%	83.3%	95.83%
	14	0.5–25	5–29	1–35	8–29	100%	100%	100%	100%	100%
*Tubakia paradryinoides*	7	0–13	6–8	12–2	0	83.3%	100%	100%	0	70.83%
	14	0–27	8–23	23–49	0	83.3%	100%	100%	0	70.83%
*Tubakia quercicola*	7	18–50	1–9	6–2	5–16	100%	100%	100%	100%	100%
	14	whole	5–17	6–22	whole	100%	100%	100%	100%	100%

## Data Availability

The sequences from the present study were submitted to the NCBI website (https://www.ncbi.nlm.nih.gov/), and the accession numbers were listed in Table 2.

## Appendix A

**Table A1:** The results of BLAST search using ITS sequence data.

Taxon	Strain	Host	GenBank Accession No.	GenBank BLAST Search Results			
				Species Identified	Strain	Accession No.	Identities (I), Query Cover (QC)
*Tubakia americana*	CFCC 55115	*Quercus acutissima*	OP114595	*T. americana*	CBS 129014	MG591873	100.00% (I), 94% (QC)
*T. americana*	CFCC 54642	*Q. aliena* var. *acuteserrata*	OP114596	*T. americana*	CBS 129014	MG591873	99.49% (I), 89% (QC)
*T. americana*	CFCC 55117	*Q. aliena* var. *acuteserrata*	OP114597	*T. americana*	CBS 129014	MG591873	100.00% (I), 94% (QC)
*T. americana*	CFCC 55980	*Q. glauca*	OP114598	*T. americana*	CBS 129014	MG591873	100.00% (I), 94% (QC)
*T. americana*	CFCC 55982	*Q. glauca*	OP114599	*T. americana*	CBS 129014	MG591873	100.00% (I), 94% (QC)
*T. americana*	CFCC 54417	*Q. variabilis*	OP114600	*T. americana*	CBS 129014	MG591873	100.00% (I), 94% (QC)
*T. americana*	CFCC 55975	*Q. glauca*	OP114601	*T. americana*	CBS 129014	MG591873	99.66% (I), 95% (QC)
*T. americana*	CFCC 56051	*Q. glauca*	OP114602	*T. americana*	CBS 129014	MG591873	99.66% (I), 95% (QC)
*T. americana*	CFCC 55970	*Q. aliena* var. *acuteserrata*	OP114603	*T. americana*	CBS 129014	MG591873	100.00% (I), 94% (QC)
*T. americana*	CFCC 54463	*Q. aliena* var. *acuteserrata*	OP114604	*T. americana*	CBS 129014	MG591873	100.00% (I), 95% (QC)
*T. americana*	CFCC 55300	*Q. aliena* var. *acuteserrata*	OP114605	*T. americana*	CBS 129014	MG591873	100.00% (I), 94% (QC)
*T. americana*	CFCC 56053	*Q. aliena* var. *acuteserrata*	OP114606	*T. americana*	CBS 129014	MG591873	100.00% (I), 95% (QC)
*T. cyclobalanopsidis*	CFCC 55961	*Q. glauca*	OP114638	*T. americana*	CBS 129014	MG591873	99.15% (I), 94% (QC)
*T. cyclobalanopsidis*	CFCC 55979	*Q. glauca*	OP114639	*T. americana*	CBS 129014	MG591873	99.31% (I), 98% (QC)
*T. cyclobalanopsidis*	CFCC 55973	*Q. glauca*	OP114640	*T. americana*	CBS 129014	MG591873	99.15% (I), 94% (QC)
*T. dryinoides*	CFCC 55958	*Q. glauca*	OP114607	*T. dryina*	Fs_NI_N09	KR362909	100.00% (I), 98% (QC)
*T. dryinoides*	CFCC 55983	*Q. glauca*	OP114608	*T. dryinoides*	CBS 329.75	MG591874	100.00% (I), 93% (QC)
*T. dryinoides*	CFCC 55966	*Q. glauca*	OP114609	*T. dryina*	Fs_NI_N09	KR362909	100.00% (I), 99% (QC)
*T. dryinoides*	CFCC 54949	*Q. acutissima*	OP114610	*T. dryina*	Fs_NI_N09	KR362909	100.00% (I), 99% (QC)
*T. dryinoides*	CFCC 54975	*Q. aliena* var. *acuteserrata*	OP114611	*T. dryina*	Fs_NI_N09	KR362909	99.84% (I), 99% (QC)
*T. koreana*	CFCC 55990	*Q. glauca*	OP114616	*T. dryinoides*	CBS 329.75	MG591874	100.00% (I), 94% (QC)
*T. koreana*	CFCC 55976	*Q. glauca*	OP114617	*T. dryinoides*	CBS 329.75	MG591874	100.00% (I), 92% (QC)
*T. koreana*	CFCC 56113	*Q. glauca*	OP114618	*T. dryinoides*	CBS 329.75	MG591874	100.00% (I), 95% (QC)
*T. koreana*	CFCC 54629	*Q. acutissima*	OP114619	*T. dryina*	Fs_NI_N09	KR362909	99.84% (I), 99% (QC)
*T. koreana*	CFCC 55967	*Q. glauca*	OP114620	*T. dryina*	Fs_NI_N09	KR362909	100.00% (I), 98% (QC)
*T. koreana*	CFCC 55977	*Q. glauca*	OP114621	*T. dryina*	Fs_NI_N09	KR362909	100.00% (I), 98% (QC)
*T. koreana*	CFCC 55963	*Q. glauca*	OP114622	*T. dryina*	Fs_NI_N09	KR362909	100.00% (I), 98% (QC)
*T. koreana*	CFCC 54968	*Q. acutissima*	OP114623	*T. dryinoides*	CBS 329.75	MG591874	99.83% (I), 98% (QC)
*T. koreana*	CFCC 54488	*Q. variabilis*	OP114624	*T. dryinoides*	CBS 329.75	MG591874	99.83% (I), 91% (QC)
*T. koreana*	CFCC 54477	*Q. variabilis*	OP114625	*T. dryina*	Fs_NI_N09	KR362909	99.83% (I), 99% (QC)
*T. koreana*	CFCC 55989	*Q. glauca*	OP114626	*T. dryinoides*	CBS 329.75	MG591874	100.00% (I), 95% (QC)
*T. koreana*	CFCC 55988	*Q. glauca*	OP114627	*T. dryina*	Fs_NI_N09	KR362909	100.00% (I), 99% (QC)
*T. koreana*	CFCC 54916	*C. mollissima*	OP114628	*T. dryina*	Fs_NI_N09	KR362909	100.00% (I), 98% (QC)
*T. paradryinoides*	CFCC 55984	*Q. glauca*	OP114612	*T. dryinoides*	CBS 329.75	MG591874	100.00% (I), 95% (QC)
*T. paradryinoides*	CFCC 55959	*Q. glauca*	OP114613	*T. dryinoides*	CBS 329.75	MG591874	100.00% (I), 95% (QC)
*T. paradryinoides*	CFCC 55974	*Q. glauca*	OP114614	*T. dryina*	Fs_NI_N09	KR362909	100.00% (I), 98% (QC)
*T. paradryinoides*	CFCC 55972	*Q. glauca*	OP114615	*T. dryina*	Fs_NI_N09	KR362909	100.00% (I), 98% (QC)
*T. quercicola*	CFCC 54426	*Q. aliena* var. *acuteserrata*	OP114629	*T. dryina*	CBS 129018	MG591871	98.54% (I), 99% (QC)
*T. quercicola*	CFCC 54471	*Q. aliena* var. *acuteserrata*	OP114630	*T. dryina*	CBS 129018	MG591871	98.90% (I), 99% (QC)
*T. quercicola*	CFCC 54284	*Q. aliena* var. *acuteserrata*	OP114631	*T. dryina*	41G	MZ078778	99.06% (I), 100% (QC)
*T. quercicola*	CFCC 54326	*Q. aliena* var. *acuteserrata*	OP114632	*T. dryina*	CBS 114919	MG591855	98.85% (I), 94% (QC)
*T. quercicola*	CFCC 54312	*Q. aliena* var. *acuteserrata*	OP114633	*T. dryina*	CBS 129018	MG591871	98.89% (I), 100% (QC)
*T. quercicola*	CFCC 54754	*Q. aliena* var. *acuteserrata*	OP114634	*T. dryina*	CBS 129018	MG591871	98.94% (I), 99% (QC)
*T. quercicola*	CFCC 55106	*Q. aliena* var. *acuteserrata*	OP114635	*T. dryina*	41G	MZ078778	99.22% (I), 99% (QC)
*T. quercicola*	CFCC 54912	*Q. aliena* var. *acuteserrata*	OP114636	*T. dryina*	CBS 129018	MG591871	99.21 % (I), 100% (QC)
*T. quercicola*	CFCC 54294	*Q. aliena* var. *acuteserrata*	OP114637	*T. dryina*	CBS 129018	MG591871	98.81 % (I), 100% (QC)
